# CONDISOX- continued versus discontinued oxytocin stimulation of induced labour in a double-blind randomised controlled trial

**DOI:** 10.1186/s12884-019-2461-x

**Published:** 2019-09-02

**Authors:** Sidsel Boie, Julie Glavind, Niels Uldbjerg, Jannet J. H. Bakker, Joris A. M. van der Post, Philip J. Steer, Pinar Bor

**Affiliations:** 1 Department of Obstetrics and Gynaecology, Randers Regional Hospital, Randers, Denmark; 2 Department of Obstetrics and Gynaecology, Aarhus University Hospital, Aarhus, Denmark; 3 Department of Obstetrics and Gynaecology, Amsterdam University Medical Centre, Amsterdam, The Netherlands; 4 Academic Department of Obstetrics and Gynaecology, Division of cancer Imperial College London, London, UK

**Keywords:** Induction of labour, Oxytocin, Discontinuation, Caesarean section

## Abstract

**Background:**

Oxytocin is an effective drug for induction of labour, but is associated with serious adverse effects of which uterine tachysystole, fetal distress and the need of immediate delivery are the most common. Discontinuation of oxytocin once the active phase of labour is established could reduce the adverse effects.

The objective is to investigate how the caesarean section rate is affected when oxytocin stimulation is discontinued in the active phase of labour compared to labours where oxytocin is continued.

**Methods:**

CONDISOX is a double-blind multicentre randomised controlled trial conducted at Danish and Dutch Departments of Obstetrics and Gynaecology. The first participant was recruited on April 8 2016.

Based on a clinically relevant relative reduction in caesarean section rate of 7%, an alpha of 0.05, a beta of 80%, we aim for 1200 participating women (600 in each arm).

The CONDISOX trial includes women at a gestational age of 37–42 complete weeks of pregnancy, who have uterine activity stimulated with oxytocin infusion for the induction of labour. Women are randomised when the active phase of labour becomes established, to study medication containing either oxytocin (continuous group) or placebo (discontinued group) infusion. Women are stratified by birth site, indication for oxytocin stimulation (induction of labour, prelabour rupture of membranes) and parity (nulliparous, parous +/− previous caesarean section).

We will compare the primary outcome, caesarean section rate, in the two groups using a chi-square test with a *p*-value of 0.05. If superiority is not demonstrated, we have a pre-defined post hoc non-inferiority boundary (margin, delta) at 1.09.

Secondary outcomes include duration of the active phase of labour, incidence of uterine tachysystole, postpartum haemorrhage, admission to the neonatal intensive care unit, Apgar score, umbilical arterial blood pH, and birth experience.

**Discussion:**

The high frequency of oxytocin use and the potential risks of both maternal and fetal adverse effects of oxytocin emphasise the need to determine the optimal oxytocin regime for induction of labour.

**Trial registration:**

NCT02553226 (registered September 17, 2015). Eudra-CT number: 2015–002942-30.

**Electronic supplementary material:**

The online version of this article (10.1186/s12884-019-2461-x) contains supplementary material, which is available to authorized users.

## Background

Syntocinon® (synthetic oxytocin) is one of the most widely used medications in obstetrics for induction of labour. In Denmark 2018, 27% nulliparous (8065 of 29,414) and 21% parous women (6636 of 31,502) had labour induced. Nearly half of these women received oxytocin stimulation as a single treatment method, or in combination with other methods (http://end2019.esundhed.dk/sundhedsregistre/MFR/Sider/MFR06A.aspx). Despite the extensive use of oxytocin only a few studies have focused on the duration of the infusion. There is no consensus as to whether oxytocin should be continued until delivery or discontinued after the onset of the active phase of labour [1–4].

The current regimen for induction of labour with oxytocin described in the national Danish Society of Obstetrics and Gynaecology (DSOG) guidelines [5] recommends that the infusion continues until delivery, unless complications (e.g. uterine tachysystole) occur, at which point the infusion rate is reduced or discontinued.

Although oxytocin is used in a high proportion of labours, its use is associated with adverse effects. The most frequent complication is uterine tachysystole [6], which increases the risk of fetal distress and birth asphyxia, requiring instrumental delivery or caesarean section. A less frequent but serious adverse event associated with the use of oxytocin is uterine rupture [7]. It is well established that oxytocin administration during labour causes a persisting down regulation of the oxytocin receptors [7], which persists postpartum and increases the risk of postpartum haemorrhage. Furthermore, initiation and duration of breastfeeding may also be adversely affected in women who undergo oxytocin stimulation [8].

We conducted a pilot study to investigate the effects of discontinued oxytocin infusion in the active phase of labour compared to continued oxytocin infusion on labour outcomes [9]. Between 2009 and 2011, two hundred women admitted for induction (188 cases) or augmentation of labour (12 cases) at the Regional Hospital of Randers were randomised to continued or discontinued oxytocin once the active phase of labour was established. The total caesarean section rate (secondary pre-specified outcome) for the oxytocin-discontinued group was 15% compared to 22% in the continued group, which was a non-significant reduction (*p* = 0.39). However, in the discontinued group, there were statistically significant fewer cases of postpartum haemorrhage, uterine tachysystole, and non-reassuring fetal heart pattern [9].

A recent published Cochrane review [10] concluded that discontinuation of oxytocin stimulation when the active phase of labour is established may reduce the caesarean section rate. However, the quality of evidence of the included trials was low, and when the analysis was restricted to results from participants who actually reached the active phase of labour, there was little or no difference on the caesarean section rate between the two groups.

### Objectives

The objective is to investigate how the caesarean section rate is affected when oxytocin stimulation is discontinued in the active phase of induced labour compared to labours where oxytocin is continued.

The incidence of other maternal and neonatal complications will be accessed as secondary outcomes.

## Methods

CONDISOX is a double-blinded multicentre randomised controlled trial conducted at Danish and Dutch Departments of Obstetrics and Gynaecology with approximately 24,000 annual births.

### Sites

#### Denmark

1. Department of Obstetrics and Gynaecology

Randers Regional Hospital

Local investigator: Sidsel Boie

2. Department of Obstetrics and Gynaecology, Aarhus University Hospital, Skejby

Local investigator: Lone Hvidman

3. Department of Obstetrics and Gynaecology, Sygehus Lillebælt, Kolding

Local investigator: Mohammad Khalil

4. Department of Obstetrics and Gynaecology, Aalborg University Hospital

Local investigator: Attila Bothazi

5. Department of Obstetrics and Gynaecology Regional Hospital Herning

Local investigator: Iben Sundtoft

6. Department of Obstetrics and Gynaecology Hillerød Regional Hospital

Local Investigator: Nini Møller

7. Department of Obstetrics and Gynaecology Rigshospitalet, Copenhagen

Local investigator: Kristina Renault

8. Department of Obstetrics and Gynecology, Odense University Hospital

Local investigator: Maja Thode Rask

9. Department of Obstetrics and Gynecology, Hvidovre Hospital

Local investigator: Lene Huusom

#### The Netherlands

1. Department of Obstetrics and Gynaecology, academic medical Centre, Amsterdam

Local investigator: Jannet Bakker.

Further centres will be included. An updated list is always available in ClinicalTrials.Gov.

#### Study dates

The first participant was recruited the 8th of April 2016. The anticipated date of recruitment completion is February 2020.

#### Participants

The CONDISOX trial will include women at 37–42 complete weeks of gestation stimulated with oxytocin infusion for induction of labour (with or without cervical priming by prostaglandin).

Exclusion criteria are as follows:

- Age < 18 years
- Unable to give written informed consent
- Cervical dilation more than 4 cm when stimulation is initiated
- Multiple pregnancies
- Non-vertex presentation
- Persistent pathological cardiotocography (CTG) before oxytocin infusion
- Estimated fetal weight of more than 4500 g.

**Fig. 1:**
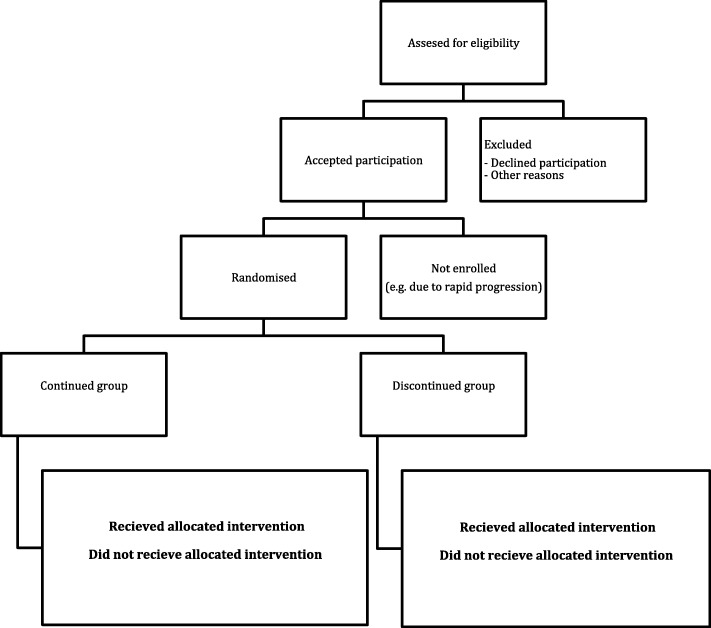
Flowchart

Figure 1 shows the study flow as outlined by the CONSORT (CONsolidated Standards Of Reporting Trials).

### Oxytocin stimulation protocol

Standard procedures will be followed prior to stimulation [11, 12]

Stimulation will be given according to national Danish [5] and Dutch [13] guidelines. The guidelines and the obstetrical care are comparable between the two countries. Intravenous infusion of 10 IU Syntocinon® diluted in 1000 ml of isotonic saline (Denmark) or 5 IU Syntocinon® diluted in 50 ml of isotonic saline (The Netherlands) is initiated at 3.3 mIU/minute and increased every 20 min by 3.3 mIU/minute until regular contractions (three to five contractions every 10 min) are achieved. The maximal dose of infusion is 30 mIU/minute.

Women will be included in the study when the active phase of labour becomes established. The definition of the active phase of labour is in accordance with latest ACOG guideline [14]: complete effacement, cervical dilatation ≥6 cm, ≥3 contractions per 10 min, and rupture of membranes. Randomisation will be performed, and the infusion will be replaced by the study medication, which will be either *Syntocinon®* at the same concentration, or a placebo infusion with saline:

1. Denmark: Continued group; 10 IE *Syntocinon®* diluted in 1000 ml 0.9% NaCl infusion; discontinued group; 1 ml 0.9% NaCl diluted in 1000 ml 0.9% NaCl infusion. (prepared by the pharmacy in ampoules identified only by study number)
2. The Netherlands: Continued group; 5 IU Syntocinon® diluted in 50 ml 0.9% NaCl infusion, discontinued group; 50 ml 0.9% NaCl. (Infusions are prepared by the pharmacy and identified only by study number).

### Outcome measures

#### Primary outcome

- Caesarean section rate

#### Secondary outcomes

- Maternal: Mode of delivery, indication for caesarean section or instrumental delivery, duration of the active phase of labour (from time of randomisation to delivery), total duration of labour from time of initiation of oxytocin stimulation until delivery, duration of time from admission to the delivery ward to delivery, incidence of uterine tachysystole, use of epidural analgesia, the total dosage of and duration of oxytocin infusion, rate of pyrexia during labour (defined as ≥38.2 **°**C with epidural, without epidural: ≥38 **°**C), rate of 3rd and 4th degree perineal tears, rate of uterine rupture, estimated volume of blood loss at delivery and postpartum, postpartum blood transfusion, the need for evacuation of retained placenta, use of antibiotics during labour, postpartum infection (defined as two confirmed maternal temperatures of ≥38 °C at least 4 h apart), urinary retention.
- Fetal/Neonatal: Death, non-reassuring fetal heart rate pattern in labour, birth weight, fetal scalp pH values, Apgar score at 1 and 5 min, umbilical cord arterial pH and blood gas values, antibiotic treatment, hyperbilirubinaemia, rate of admission to neonatal intensive care unit (NICU), or any need for resuscitation (bag and mask or intubation, time to onset of spontaneous ventilation).
- Breastfeeding (successful establishment and duration of exclusive breastfeeding)
- Birth experience and patient satisfaction 4 weeks postpartum (Childbirth Experience Questionnaire, CEQ1 [15])

All outcome data will be registered in an eCRF (see Additional file 1) designed for the trial using the REDCap database (including range checks for data values and double entry for primary outcome and selected secondary outcomes). The data collection form can be obtained by contacting the corresponding author.

For participants who discontinue or deviate from the trial protocol all of the above outcomes are still to be collected if available.

### Randomisation, blinding and informed consent

Women will be informed of the trial when induction of labour is planned or at the first visit to the labour ward in case of prelabour rupture of membranes. Subjects’ signed informed consent to participation will be obtained prior to randomisation and intervention (i.e. before any oxytocin stimulation).

Women will be randomised in a 1:1 ratio to either the continued oxytocin group or the discontinued oxytocin group using an Internet-based randomisation programme (Trialpartner). Random block-sizes of four will be used, and the women will be stratified by site, parity (nulliparous or parous +/− previous caesarean) and indication for oxytocin infusion (induction of labour or induction due to prelabour rupture of membranes (PROM)). The randomisation number generated by the computer programme corresponds to number of the studymedication (masked, identical ampoules). The personnel of the delivery ward will administer the medication according to existing guidelines concerning administration [5, 12, 13]. Women, caregivers, and trialmanagers will be blinded to the allocation due to the use of identical study medication preparations.

### Complications

In accordance with guidelines [5, 13] The infusion will be reduced or discontinued at any point of labour, if the following occur:

- Tachysystole (> 5 contractions per 10 min, averaged over a 30-min window)
- Uterine contractions lasting 2 min or more
- Non-reassuring CTG (recurrent variable decelerations, fetal tachycardia or bradycardia, minimal to absent baseline variability, late decelerations) or significant STAN event(s)
- Suspicion of uterine rupture

### Dystocia

If there is failure to progress, defined as less than two cm dilation over 4 h despite apparently adequate contractions, and/or maximal infusion rates (oxytocin or placebo), the study medication can be replaced with open-labelled oxytocin infusion.

If failure to progress persists, despite open-labelled oxytocin infusion for 4 h, caesarean section can be considered.

### Side effects and risks

We expect a rate of persistent failure to progress of 8–46% among the participants in the discontinued group versus 3–17% in the continued group [1–4]. Based on data from the pilot study [9], we expect a caesarean section rate of 15% in the discontinued group versus 22% in the continued group. According to the pilot study and previous studies [1–4], maternal and neonatal complications in the discontinued group are expected to be lower than in the continued group (The 2018 data for labour that are induced in Denmark indicate an acute caesarean section rate of 14.6%. However, the latter figure includes women who respond to prostaglandin alone and there are no data for women who require oxytocin infusion in addition).

All women will be monitored with continuous electronic fetal heart rate monitoring during labour to detect complications such as uterine tachysystole and non-reassuring/pathological fetal heart rate in accordance with national guidelines. Women and their newborns will be observed for at least 3–6 h postpartum (termination of study medication) according to the current practice on the delivery ward before discharged home.

Delivery ward staff is responsible for timely reporting of any adverse reactions to the trial manager.

Adverse reactions/events will be registered immediately in the electronic medical file of the patient. The Summary of Product Characteristics (SPC) of *Syntocinon®* will be used as reference [6] to determine whether a Serious Adverse Reaction is expected or unexpected. The primary investigator or a nominated deputy will go through the participants’ electronic medical record 7–30 days postpartum during data collection. The primary investigator will ensure that all relevant information about suspected serious unexpected adverse reactions that are fatal or life threatening is recorded. The primary investigator will report as soon as possible (and in any case no later than seven days) to the competent authorities concerned. The primary investigator will ensure that any relevant follow-up information is subsequently communicated within an additional eight days.

The primary investigator will report to the competent authorities concerned and to the Ethics Committees all other suspected unexpected serious adverse reactions as soon as possible but within a maximum of 15 days of first knowledge.

### Power calculation and statistical analysis

Based on the pilot study [9] we expect a caesarean section rate of 22% in the continued group. A clinically relevant relative reduction in the caesarean section rate would be 30%, corresponding to a caesarean section rate of 15% in the discontinued group. Aiming for a power (beta) of 80% and an alpha of 0.05, superiority can be shown with a sample size of 482 women in each group. Allowing for crossover and a dropout of 5% we aim to recruit a total of 600 women per treatment arm (1200 in total).

If superiority of oxytocin discontinuation vs. continued oxytocin stimulation for the reduction of the incidence of caesarean section cannot be shown, non-inferiority testing is a relevant alternative. It is plausible that improvements in secondary outcomes will be seen, even if there is no superiority on the primary outcome. To allow formal non-inferiority testing as an alternative, we define a post hoc non-inferiority boundary (margin, delta) at 1.09. This boundary is to exclude a rate of 22% in the continued oxytocin group as compared to 24% in the discontinued group.

Data will be analysed according the intention-to-treat principle. Basic demographic data will be presented with counts and percentages for categorical variables, mean and standard deviation for continuous Gaussian distributed variables, and median and interquartile range for continuous non-Gaussian variables. The primary outcome variable will be assessed by comparing the event rates in the two groups using a chi-square test with a significance threshold *p*-value of < 0.05. Results will be presented as absolute and relative risks along with 95% confidence intervals (Cl) and the numbers needed to treat (if applicable). Categorical secondary outcomes will be assessed in the same way as the primary outcome. For continuous secondary outcomes with a Gaussian distribution (following log transformation if appropriate) we will assess differences between groups using the student’s t-test, and with a non-parametric Mann-Whitney U test if the data are non-Gaussian. We will present time to delivery using Kaplan-Meier estimates and survival curves, and test the differences between the two groups using the log-rank test. We will use multivariate logistic regression with adjustment for indifferences in baseline characteristics to calculate odds ratios with 95% confidence intervals.

Subgroup analysis will be undertaken for the following subgroups:

- Indication for stimulation (PROM and induction)
- Parity (nulliparous and parous)
- Previous caesarean section

### Monitoring

The trial is continuously monitored according to Good Clinical Practice (GCP). Each recruiting site is visited at least once per year by the external monitor who performs an audit on selective outcome measures.

An interim analysis is performed each year during the inclusion period. Three independent members have been assigned to the data monitoring committee (DMEC, see Additional file 2) and they have access to the results of the interim analysis. DMEC members safeguard the interests of trial participants, assess the safety of the intervention during the trial, and monitor the overall conduct of the clinical trial. No formal stopping rules have been made. A report is sent to the Trial Steering Committee (TSC, see Additional file 3) on the conclusion of the assessment made by the DMEC. The TSC provides independent advice to the trialists based on the DMEC conclusion.

The charter for the DMEC and/or the TSC can be obtained by contacting the corresponding author of the trial.

In case of important protocol changes, the amendment will be communicated to the relevant parties (site investigators, trial registry etc.).

## Discussion

The high frequency of oxytocin use and the potential risks of both maternal and fetal adverse effects of oxytocin emphasise the need to determine the optimal oxytocin regime for induction of labour. Adverse effects of oxytocin are associated with considerable socio-economic and human costs. Reducing the duration of oxytocin stimulation during labour may reduce the risk of acute caesarean section, the number of newborn with asphyxial sequelae and the number of maternal and neonatal adverse events during labour and delivery.

## Additional files

Additional file 1: Electronic case record form eCRF. (PDF 106 kb)

Additional file 2: Data Monitoring and Ethics Committee. (DMEC) (DOCX 39 kb)

Additional file 3: Trial Steering Committee (TSC). (DOCX 51 kb)

## Data Availability

The trial is on going. When the primary data from the trial are published the data will become publicly available. The results will be presented in international peer reviewed journals and at relevant international conferences.

## Abbreviations

Amsterdam UMC: Amsterdam University Medical Centre
CEQ1: Childbirth Experience Questionnaire 1
CI: Confidence Interval
CONSORT: Consolidated Standards of Reporting Trials
CTG: Cardio Toco Graphy
DMEC: Data Monitoring and Ethics Committee
NICU: Neonatal Intensive Care Unit
PROM: Prelabour Rupture Of Membranes
SPC: Summary of Product Characteristics
STAN: ST-analysis
TSC: Trial Steering Committee
