# Supplementary material for: CONDISOX- continued versus discontinued oxytocin stimulation of induced labour in a double-blind randomised controlled trial
Source: BMC Pregnancy Childbirth. 2019 Sep 2;19:320. doi: 10.1186/s12884-019-2461-x (PMC6720847; doi:10.1186/s12884-019-2461-x)
Supplement: Supplementary file 1 — Electronic case record form eCRF. (PDF 106 kb) [file 12884_2019_2461_MOESM1_ESM.pdf]

Inclusion

Patient ID ______________________

Civil Registration Number (CPR-nr) ______________________

Birthcenter
- ◯ Aarhus
- ◯ Randers
- ◯ Kolding
- ◯ Aalborg

Parity
- ◯ nullipara
- ◯ multipara UDEN tidl. sectio
- ◯ multipara MED tidl.sectio

Indication
- ◯ Induction
- ◯ PROM (Premature Rupture of Membranes)

Treatment
- ◯ Placebo
- ◯ Syntocinon

Randomization number ______________________

Package number ______________________

Randomization date ______________________

Kodebruds initialer ______________________

Kodebruds tekst

____

Kodebruds dato ______________________

Contact information (for questionaires)

e-mail adress ______________________________

Paticipant caracteristics and history

Parity

◯ 0 - nullipara
◯ 1 - primipara
◯ 2
◯ 3
◯ 4
◯ 5
◯ 6
◯ 7
◯ Other
◯ NA
(Choose the number corresponding to the parity of the participant prior to the current pregnancy and labour.)

If other, enter parity

____
(IN case of missing value enter 99)

Prior caesarean section

◯ Yes
◯ No
◯ NA
(State if the women have had a Caesarean section prior to the current delivery. If no data available choose NA)

Height

____
(Enter the height of participant in centimeter (cm). If missing value please state: 999)

Weight prior to current pregnancy

____
(Enter weight of the participant in kilograms (kg) without decimals prior to pregnancy. If missing value please state 999)

BMI

____
((calculation field))

Educational status

◯ No schooling/less than primary
◯ Primary
◯ Lower secondary
◯ Upper secondary
◯ Post secondary non-tertiary
◯ Short cycle tertiary
◯ Bachelor or equivalent
◯ Master or equivalent
◯ Doctoral or equivalent
◯ Other
◯ NA
(In case of missing value choose NA)

If Other, enter education

____

Etnicity

◯ Caucasian
◯ Asian
◯ Afro-Caribbean
◯ Oriental
◯ Other
◯ Unknown
(Choose one box to indicate the race of the participant)

If other, enter race
____
(In case of missing value enter NA)

Smoking status during pregnancy
◯ Ever
◯ Never
◯ NA
(If missing value please state: NA)

marital status
◯ single
◯ maried
◯ co-habiting
◯ NA
(In case of missing value choose NA)

Estimated birth weight
____
(Enter the latest estimated birth weight in gram. If no data available enter 9999)

Birth weight estimation method
☐ Ultrasound
☐ Clinical examination
☐ NA
(Enter how the birth weight estimate has been done. If missing value please choose NA)

Which medical condition(s) of the below listed did the woman suffer from during pregnancy and labour?
☐ Gestational Diabetes
☐ Diabetes Mellitus 1
☐ Diabetes Mellitus 2
☐ Hypertension
☐ Preeclampsia
☐ Metabolic disease
☐ Intrauterine Growth Restriction (IUGR)
☐ Autoimmune disease
☐ Depression (treated with Anti-depressives at time of labour)
☐ None of the above
(Enter all of the conditions listet above that the woman suffers from)

Gestational age at birth (number of whole weeks)
____
(Enter the number of gestational age in whole weeks of pregnancy, e.g. GA 37+5 is to be entered as 37. In case of missing value enter 99)

Gestational age at birth (number of days in incompleted week)
____
(Enter the number of days in incompleted week of gestational age. E.g. GA 37+5 is to be entered as 5. If missing value enter 99)

Gestational age in days
____
(Calculated field. Will be filled out automatically)

Prostaglandine prior to stimulation with oxytocin
◯ Yes
◯ No
◯ NA
(Enter whether the woman has recieved prostaglandine (misoprostol/Angusta) prior to stimulation with oxytocin. If data is missing choose NA)

Total dosage of prostaglandine
____
(Enter total dosage of prostaglandine administered in mikrogram. If missing value enter 999)

Balloon dilatation prior to stimulation with oxytocin

◯ yes
◯ No
◯ NA
(Choose if a cook cervical ripening baloon catheter has been used for mechanical dilatation of the cervical canal prior to labour induction. In case of missing value choose NA)

Start of labour (Stimulation protocol)

Admission date and time on the delivery ward
____
(If missing value enter 01-01-2001 00:00)

Primary indication for stimulation with oxytocin
◯ PROM (Premature Rupture Of Membranes)
◯ Diabetes
◯ Gravida Prolongata
◯ Hypertension/preeclampsia
◯ IUGR (IntraUterine fetal Growth Restriction)
◯ Maternal request
◯ BMI >35
◯ Oligohydramnios
◯ Cholestasis
◯ NA
(Choose the primary indication for stimulation with oxytocin. If data is missing choose NA)

Date and time for initiation of stimulation with oxytocin
____
(If missing value enter 01-01-2001 00:00)

Cervical dilatation in centimeters (cm) at initiation time of stimulation with oxytocin
____
(If missing value enter 9)

Rupture of membranes or amniotomy prior to stimulation with oxytocin
◯ Yes
◯ No
◯ NA
(If missing value choose NA)

Date and time for rupture of membranes
____
(If missing value enter 01-01-2001 00:00)

Description of amniotic fluid
◯ clear
◯ meconium stained
◯ NA
(If missing value choose NA)

Use of epidural analgesia
◯ Yes
◯ No
◯ NA
(If missing value choose NA)

Date and time for insertion of epidural analgesia
____
(In case of missing value 01-01-2001 00:00)

Total number of vaginal examinations during stimulation with oxytocin, prior to project medicine
◯ 1
◯ 2
◯ 3
◯ 4
◯ 5
◯ 6
◯ NA
(If missing value choose NA)

Pausation of oxytocin prior to commencement of project medicine
◯ Yes
◯ No
◯ NA
(If missing value choose NA)

Time for pausation
____
(If missing value enter 01-01-2001 00:00)

Reason for pausation of oxytocin	☐ Uterine hyperstimulation<br>☐ Non-reasurrent CTG<br>☐ NA<br>(If missing value enter NA)
Total given volumen oxytocin	__________________________________<br>(If missing value enter 9999)
Initiation date and time for project medicine	__________________________________<br>(If missing value enter 01-01-2001 00:00)
Cervical dilatation at initiation time for project medicine<br>in centimeters (cm)	__________________________________<br>(If missing value enter 99)
Project medicine discontinued earlier than guideline provides (prior to delivery of the placenta)	◯ Yes<br>◯ No<br>◯ NA<br>(If missing value choose NA)
Time for early discontinuation of project medicine	__________________________________<br>(If missing value enter 01-01-2001 00:00)
Reason for early discontinuation of project medicine	☐ Uterine hyperstimulation<br>☐ Non-reasurrent CTG<br>☐ Dystocia<br>☐ Other<br>☐ NA<br>(If missing value please enter NA)
Resumption of project medicine	◯ Yes<br>◯ No<br>◯ NA<br>(If missing value choose NA)
Time for resumption of the project medicine	__________________________________<br>(If missing value enter 01-01-2001 00:00)
End time for stimulation with project medicine	__________________________________<br>(If missing value enter 01-01-2001 00:00)
Duration of project medicine (minutes)	__________________________________<br>(calculated field. Will be filled out automatically)
Maximal dosage of project medicine (mL/hour)	__________________________________<br>(If missing value enter 999)
Duration of stimulation in total	__________________________________<br>(Calculated field. Will be filled out automatically)
Total given volumen projectmedicin (ml)	__________________________________<br>(If missing value enter 9999)
Violation of the project protocol?	◯ Yes<br>◯ No<br>◯ NA<br>(If missing value choose NA)
Describe any violance of the trial protocol	____

BLINDING

Unconcealed allocation of participant to personnel

- ◯ Yes

Reason for unconcealment

- ◯ maternal request
- ◯ physician request
- ◯ midwife request
- ◯ NA

(If missing value enter NA)

Course of labour 1

Uterine hypertonia (single contraction lasting > 2 minutes) prior to project medicine	◯ Yes<br>◯ No<br>◯ NA<br>(DURING STIMULATION WITH OXYTOCIN If missing value choose NA)
Uterine hyperstimulation prior to project medicine?<br><br>Uterine hyperstimulation is when stimulation with oxytocin leads to >5 uterine contactions per 10 minutes, during a period of 30 minutes.	◯ Yes<br>◯ No<br>◯ NA<br>(DURING STIMULATION WITH OXYTOCIN If missing value choose NA)
Worst CTG classification during stimulation with oxytocin prior to project medicine	◯ Normal<br>◯ Suspicious<br>◯ Patological<br>◯ Preterminal<br>◯ NA<br>(DURING STIMULATION WITH OXYTOCIN. If missing value choose NA)
Uterine hypertonia (single contraction lasting > 2 minutes) during project medicine	◯ Yes<br>◯ No<br>◯ NA<br>(DURING STIMULATION WITH PR0OJECTMEDICINE. If missing value choose)
Uterine hyperstimulation during project medicine?<br><br>Uterine hyperstimulation is when stimulation with oxytocin leads to >5 uterine contactions per 10 minutes, during a period of 30 minutes.	◯ Yes<br>◯ No<br>◯ NA<br>(DURING STIMULATION WITH PR0OJECTMEDICINE. If missing value choose)
Worst CTG classification during treatment with project medicine	◯ Normal<br>◯ Suspicious<br>◯ Patological<br>◯ Preterminal<br>◯ NA<br>(DURING STIMULATION WITH PR0OJECTMEDICINE.If missing value choose NA)
Fetal-scalp blood measurement(s) during labour	◯ Yes<br>◯ No<br>◯ NA<br>(If missing value choose NA)

Total number of fetal-scalp blood measurements

◯ 1
◯ 2
◯ 3
◯ 4
◯ 5
◯ 6
◯ 7
◯ 8
◯ 9
◯ 10
◯ 11
◯ 12
◯ NA

(If 2 measurements routinely are performed at the same time, this only counts as 1 scalp pH measure. If missing value choose NA)

Lowest measured fetal-scalp pH value

____
(Enter 9, if no meassurement has been done)

Lowest measured fetal-scalp lactate value

____
(Enter 9, if no meassurement has been done)

Course of labour 2

Date and time for initiation of second stage of labour
____
(Enter the time when dilatation of cervix is 10 cm. If missing value enter 01-01-2001 00:00)

Initiation date and time for the active pushing periode
____
(If missing value enter 01-01-2001 00:00)

Time and date of birth
____
(If missing value enter 01-01-2001 00:00)

Duration of labour (minutes)
(stimulation with oxytocin until birth)
____
(Calculated field. Will be filled out automatically)

Date and time for discontinuation of stimulation (projectmedicin or oxytocin)
____
(If missing value enter 01-01-2001 00:00)

If project medicine replaced with open labelled oxytocin:
Total volumen of oxytocin given
____
(If missing value or non given: enter 99999)

liveborn
- ◯ Yes
- ◯ No
- ◯ NA

(If missing value choose NA)

Date and time for intrauterine death
____
(If missing value enter 01-01-2001 00:00)

Mode of delivery
- ◯ Vaginal
- ◯ Vacuum extraction
- ◯ Ceasarean
- ◯ Forceps
- ◯ NA

(If missing value choose NA)

Indication for instrumental or caesarean delivery
- ◯ Asphyxia
- ◯ Tired mom
- ◯ dystocia
- ◯ suspicion of uterine rupture
- ◯ suspicion of chorioamnionitis
- ◯ other
- ◯ NA

(If missing value choose NA)

Describe other
____

Urgency of Ceasarean section
- ◯ Grade 1 (Acute: immediate delivery)
- ◯ Grade 2 (Delivery within 30 minutes from decision time)
- ◯ Grade 3 (Delivery within 60 minutes from decision time)
- ◯ NA

(Choose one of the choices above. If missing value please choose NA)

Cervical dilatation in centimeters (cm) at time of ceasarean section
____
(If missing value state 99)

Uterine rupture	◯ Yes<br>◯ No<br>◯ NA<br>(If missing value please choose NA)
Intrapartum fever<br><br>With epidural: >38.2 degrees celsius<br>Without epidural: >38 degrees celsius	◯ Yes<br>◯ No<br>◯ NA<br>(In case of missing value choose NA)
Time for registration of intrapartum fever	______________________________<br>(In case of missing value enter 01-01-2001 00:00)

Postpartum period

Did the woman incur a perineal tear

◯ Yes
◯ No
◯ NA
(In case of missing value choose NA)

Degree of perineal tear:

Grade 1 (include only perineum)
Grade 2 (include also perineal muscle)
Grade 3a (include < 50% of sphinchter ani)
Grade 3 b (include > 50% of sphincter ani)
Grade 4 (include sphinchter ani and mucosa of rectum)

◯ Grade 1
◯ Grade 2
◯ Grade 3a
◯ Grade 3b
◯ Grade 4
◯ NA
(If missing value choose NA)

Was the placenta removed manually?

◯ Yes
◯ No
◯ NA
(In case of missing value choose NA)

Volumen in mililiters (ml) of blood loss during delivery and the first 24 hours postpartum

____
(In case of missing value state 9999)

Given treatment for postpartum hemorrhage

☐ Bimanual uterine massage
☐ Medical
☐ Bakri-ballon
☐ B lynch
☐ Bloodtransfusion
☐ Profylactic Syntocinon (intramuscular injection)
☐ other
☐ No treatment needed/given
☐ NA
(State the given treatment for atonia)

Describe other

____

Did urinary retention postpartum ocure?

Defined by:
1.No spontanously urination 6 hours postpartum if vaginal delivery
2. No spontaneously urination 6 hours after removal of CAD (catheter á demeure) if caesarean delivery

◯ Yes
◯ No
◯ NA
(In case of missing value choose NA)

Treatment for urinary retention

☐ Intermittent Catheterisation with disposable catheter
☐ Catheter á demeure
☐ NA
(If missing value choose NA)

Time for treatment for urinary retention

____
(If missing value enter 01-01-2001 00:00)

Antibiotic treatment due to puerperal infection

◯ Yes
◯ No
◯ NA
(If missing value choose NA)

Date and time for discharge of the woman

____
(If missing value enter 01-01-2001 00:00)

Duration of admission

____
(Calculated field. Will be filled out automatically)

Readmission within the first week postpartum

◯ Yes
◯ No
◯ NA
(In case of missing value choose NA)

Readmission due to

☐ Suspected infection
☐ Endometritis proven with culture
☐ Urinary tract infection treated with antibiotics
☐ Wound infection treated with abtibiotics
☐ Bowel obstruction
☐ Pneumonia
☐ Trombo-embolic complications
☐ Eclampsia
☐ HELLP
☐ Admission due to child, no maternal reason
☐ Other
☐ NA
(If missing value choose NA)

Serious Adverse Events

Maternal cardic arrest during labour

◯ Yes
◯ No
◯ N/A
(In case of missing value choose NA)

Maternal amniotic fluid emboli

◯ Yes
◯ No
◯ N/A
(If missing value enter NA)

Maternal thromboembolic event

◯ Yes
◯ No
◯ NA
(In case of missing value choose NA)

Maternal Pulmonary edema

◯ Yes
◯ No
◯ N/A
(In case of missing value choose NA)

Neonate + breastfeeding

Civil registration number (CPR nr) of newborn | ____________________ (10 numbers without using hyphen or space)

Birth weight in gram | ____________________ (In case of missing value enter 9999)

Apgar score at 1 minute | ____________________ (If missing value enter 99)

Apgar score at 5 minutes | ____________________ (. If missing value enter 99)

Apgar score at 10 minutes | ____________________ (If missing value enter 99)

Umbilical cord measurements

Umbilical artherial blood pH | ____________________ (If missing value enter 99)

Umbilical artherial blood SBe (Standard Base excess) | ____________________ (If missing value enter 99)

Date and time of birth of placenta | ____________________ (. If missing value enter 01-01-2001 00:00)

Duration between birth of neonate and birth of placenta | ____________________ (Calculated field. Will be filled out automatically)

Neonate licking with in the first 2 hours post partum
- ◯ Yes
- ◯ No
- ◯ NA

(In case of missing value please choose NA)

Neonate sucking within the first 2 hours postpartum
- ◯ Yes
- ◯ No
- ◯ NA

(In case of missing value choose NA)

Nutrition at discharge
- ◯ Exclusive breastfeeding
- ◯ Not exclusive breastfeeding
- ◯ NA

(In case of missing value choose NA)

Neonatal resuscitation within the first 10 minutes postpartum
- ◯ Yes
- ◯ No
- ◯ NA

(If missing value please choose NA)

Was the newborn ventilated with in the first hour postpartum
- ◯ Yes
- ◯ No
- ◯ NA

(If missing value choose NA)

Did the newborn recieve CPAP (Continuous Pulmonary Airway pressure) treatment during the first 24 hours postpartum?	◯ Yes<br>◯ No<br>◯ NA<br>(If missing value choose NA)
Intubation of the neonate within the first 24 hours postpartum	◯ Yes<br>◯ No<br>◯ NA<br>(If missing value choose NA)
Neonatal death within 7 days postpartum	◯ Yes<br>◯ No<br>◯ NA<br>(If missing value choose NA)
Date and time of death	__________________________________<br>(Ved missing value angiv 01-01-2001 00:00)
Was the newborn admitted to Neonatal Intensive Care Unit (NICU) within the first 24 hours	◯ Yes<br>◯ No<br>◯ NA<br>(If missing value choose NA)
ICD 10 codes (diagnosis-code) during admission at the Neonatal Intensive Care Unit (NICU)	__________________________________<br>(Enter the codes registered in the medical record of the newborn during the admission in NICU. If missing value enter NA)
Highest bilirubin-value measured	__________________________________<br>(If missing value enter 9999)
Enter the time for bilirubin meassurement	__________________________________<br>(If missing value enter 01-01-2001 00:00)
Did the newborn recieve therapy due to hyperbilirubiaemia	◯ Yes<br>◯ No<br>◯ NA<br>(If missing value please choose NA)

Long term maternal: Breastfeeding

Exclusive breastfeeding 17 weeks

◯ Yes
◯ No
◯ NA
(Data from Børnedatabasen. In case of missing value enter NA)

Din fødselsoplevelse

Et af mlene for Condisox-projektet er en positiv fdslsoplevelse for kvinder, som modtager ve-stimulerende drop.

Formlet med dette sprgeskema er at undersge, hvordan du oplevede fdslen.
Vi har valgt at anvende et internationalt anerkendt sprgeskema (CEQ- Childbirth Experience Questionaire).

Dine svar er vigtige for vores projekt og for kvinder som i fremtiden skal modtage vestimulerende drop. Derfor vil vi bede dig svare p alle sprgsml. Nogle af sprgsmlene passer mske ikke helt p dig. Vi vil gerne du have, du vlger de svar, der passer s godt som muligt p dig og din fdselsoplevelse.

Det tager ca 7 minutter at udfylde sprgeskemaet.

133) Fødslen forløb som jeg havde forestillet mig.

- ◯ Passer helt
- ◯ Passer delvist
- ◯ Passer ikke særlig godt
- ◯ Passer slet ikke
- ◯ Ønsker ikke at svare

(Sæt kryds i én boks)

134) Jeg følte mig stærk under fødslen.

◯ Passer helt
◯ Passer delvist
◯ Passer ikke særlig godt
◯ Passer slet ikke
◯ Ønsker ikke at svare
(Sæt kryds i én boks)

135) Jeg følte mig bange under fødslen.

◯ Passer helt
◯ Passer delvist
◯ Passer ikke særlig godt
◯ Passer slet ikke
◯ Ønsker ikke at svare
(Sæt kryds i én boks)

136) Jeg følte mig i stand til at gennemføre fødslen.

◯ Passer helt
◯ Passer delvist
◯ Passer ikke særlig godt
◯ Passer slet ikke
◯ Ønsker ikke at svare
(Sæt kryds i én boks)

137) Jeg var træt under fødslen.

◯ Passer helt
◯ Passer delvist
◯ Passer ikke særlig godt
◯ Passer slet ikke
◯ Ønsker ikke at svare
(Sæt kryds i én boks)

138) Jeg var glad under fødslen.

◯ Passer helt
◯ Passer delvist
◯ Passer ikke særlig godt
◯ Passer slet ikke
◯ Ønsker ikke at svare
(Sæt kryds i én boks)

139) Jeg har mange positive minder fra fødslen.

◯ Passer helt
◯ Passer delvist
◯ Passer ikke særlig godt
◯ Passer slet ikke
◯ Ønsker ikke at svare
(Sæt kryds i én boks)

140) Jeg har mange negative minder fra fødslen.

◯ Passer helt
◯ Passer delvist
◯ Passer ikke særlig godt
◯ Passer slet ikke
◯ Ønsker ikke at svare
(Sæt kryds i én boks)

141) En del af minderne fra fødslen kan få mig til at føle mig nedtrykt.

- ◯ Passer helt
- ◯ Passer delvist
- ◯ Passer ikke særlig godt
- ◯ Passer slet ikke
- ◯ Ønsker ikke at svare

(Sæt kryds i én boks)

142) Jeg følte, at jeg havde mulighed for at påvirke, om jeg skulle være oppe og røre mig eller ligge ned.

◯ Passer helt
◯ Passer delvist
◯ Passer ikke særlig godt
◯ Passer slet ikke
◯ Ønsker ikke at svare
(Sæt kryds i én boks)

143) Jeg følte, at jeg havde mulighed for at påvirke fødselsstillingen

- ◯ Passer helt
- ◯ Passer delvist
- ◯ Passer ikke særlig godt
- ◯ Passer slet ikke
- ◯ Ønsker ikke at svare

(Sæt kryds i én boks)

144) Jeg følte, at jeg havde mulighed for at påvirke valg af smertelindring.

◯ Passer helt
◯ Passer delvist
◯ Passer ikke særlig godt
◯ Passer slet ikke
◯ Ønsker ikke at svare
(Sæt kryds i én boks)

145) Jordemoderen brugte tilstrækkelig tid på mig.

- ◯ Passer helt
- ◯ Passer delvist
- ◯ Passer ikke særlig godt
- ◯ Passer slet ikke
- ◯ Ønsker ikke at svare

(Sæt kryds i én boks)

146) Jordemoderen brugte tilstrækkelig tid på min partner.

◯ Passer helt
◯ Passer delvist
◯ Passer ikke særlig godt
◯ Passer slet ikke
◯ Ønsker ikke at svare
(Sæt kryds i én boks)

147) Jordemoderen informerede om, hvad der skete under fødslen.

◯ Passer helt
◯ Passer delvist
◯ Passer ikke særlig godt
◯ Passer slet ikke
◯ Ønsker ikke at svare
(Sæt kryds i én boks)

148) Jordemoderen forstod mine behov.

- ◯ Passer helt
- ◯ Passer delvist
- ◯ Passer ikke særlig godt
- ◯ Passer slet ikke
- ◯ Ønsker ikke at svare

(Sæt kryds i én boks)

149) Jeg følte, at jordemoderen behandlede mig godt.

◯ Passer helt
◯ Passer delvist
◯ Passer ikke særlig godt
◯ Passer slet ikke
◯ Ønsker ikke at svare
(Sæt kryds i én boks)

150) Mit indtryk af sygehuspersonalets faglige kompetence gjorde mig tryg.

◯ Passer helt
◯ Passer delvist
◯ Passer ikke særlig godt
◯ Passer slet ikke
◯ Ønsker ikke at svare
(Sæt kryds i én boks)

151) Jeg følte, at jeg håndterede situationen godt.

◯ Passer helt
◯ Passer delvist
◯ Passer ikke særlig godt
◯ Passer slet ikke
◯ Ønsker ikke at svare
(Sæt kryds i én boks)

152) Hvor smertefuldt oplevede du generelt fødslen?

◯ Ingen smerte
◯
◯
◯
◯
◯
◯
◯
◯
◯ Værst tænkelige smerte
◯ Ønsker ikke at svare
(Sæt kryds i det felt på skalaen, som passer bedst på din smerte)

153) Hvor meget kontrol følte du, at du generelt havde under fødslen?

- ◯ Ingen kontrol
- ◯
- ◯
- ◯
- ◯
- ◯
- ◯
- ◯
- ◯
- ◯ Fuldstændig kontrol
- ◯ Ønsker ikke at svare

(Sæt kryds i det felt på skalaen, som passer bedst på din oplevelse)

154) Når du tænker tilbage på fødslen, hvor tryg følte du dig generelt?

- ◯ Slet ikke tryg
- ◯
- ◯
- ◯
- ◯
- ◯
- ◯
- ◯
- ◯
- ◯ Fuldstændig tryg
- ◯ Ønsker ikke at svare

(Sæt kryds i det felt på skalaen, som passer bedst på din oplevelse)

155) Egne kommentarer

____

(Her har du mulighed for at tilføje dine egne kommentarer)
