# Supplementary material for: CONDISOX- continued versus discontinued oxytocin stimulation of induced labour in a double-blind randomised controlled trial
Source: BMC Pregnancy Childbirth. 2019 Sep 2;19:320. doi: 10.1186/s12884-019-2461-x (PMC6720847; doi:10.1186/s12884-019-2461-x)
Supplement: Supplementary file 2 — Data Monitoring and Ethics Committee. (DMEC) (DOCX 39 kb) [file 12884_2019_2461_MOESM2_ESM.docx]

Additional file 2

**DATA MONITORING AND ETHICS COMMITTEE (DMEC)**

1. Lone Krebs

Chair of DMEC, associate professor, DMSc, MD, Department of Obstetrics and Gynaecology, Holbæk Sygehus, Denmark

2. Martin Berg Johansen

Member of DMEC, Statistician, MSc, Aalborg University

3. Gorm Greisen

Member of DMEC, professor and Chair of Danish Ethics Committee, Department of Paediatrics, Rigshospitalet, Copenhagen, Denmark
