# Supplementary material for: CONDISOX- continued versus discontinued oxytocin stimulation of induced labour in a double-blind randomised controlled trial
Source: BMC Pregnancy Childbirth. 2019 Sep 2;19:320. doi: 10.1186/s12884-019-2461-x (PMC6720847; doi:10.1186/s12884-019-2461-x)
Supplement: Supplementary file 3 — Trial Steering Committee (TSC). (DOCX 51 kb) [file 12884_2019_2461_MOESM3_ESM.docx]

Additional file 3

**TRIAL STEERING COMMITTEE (TSC)**

1. Jim Thornton

Chief of TSC, professor, Division of Child Health, Obstetrics & Gynaecology, School of Medicine, University of Nottingham, UK

2. Thomas Bergholt

Independent member of TSC, associate professor, PhD, MD, Department of Obstetrics and Gynaecology Rigshospitalet, Copenhagen, Denmark

3. Wessel Ganzevoort

Independent member of TSC, PhD, MD, Department of Obstetrics and Gynaecology, Academic Medical Centre, Amsterdam, The Netherlands

4. Jens Fuglsang, Associate professor, PhD, MD, Department of Obstetrics and Gynaecology, Aarhus University Hospital, Denmark

5. Inger Stornes, MD, Department of Obstetrics and Gynaecology, Regional Hospital of Randers
